# Characterization of spatial integrity with active and passive implants in a low-field magnetic resonance linear accelerator scanner

**DOI:** 10.1016/j.phro.2024.100576

**Published:** 2024-04-07

**Authors:** Bertrand Pouymayou, Yoel Perez-Haas, Florin Allemann, Ardan M. Saguner, Nicolaus Andratschke, Matthias Guckenberger, Stephanie Tanadini-Lang, Lotte Wilke

**Affiliations:** a Department of Radiation Oncology, University Hospital Zurich and University of Zurich, Zurich, Switzerland; b Department of Traumatology, University Hospital Zurich and University of Zurich, Zurich, Switzerland; c Department of Cardiology, University Hospital Zurich and University of Zurich, Zurich, Switzerland

**Keywords:** Implants, Distortion, MR-Linac, MR-guided radiotherapy

## Abstract

**Background and Purpose:**

Standard imaging protocols can guarantee the spatial integrity of magnetic resonance (MR) images utilized in radiotherapy. However, the presence of metallic implants can significantly compromise this integrity. Our proposed method aims at characterizing the geometric distortions induced by both passive and active implants commonly encountered in planning images obtained from a low-field 0.35 T MR-linear accelerator (LINAC).

**Materials and Methods:**

We designed a spatial integrity phantom defining 1276 control points and covering a field of view of 20x20x20 cm^3^. This phantom was scanned in a water tank with and without different implants used in hip and shoulder arthroplasty procedures as well as with active cardiac stimulators. The images were acquired with the clinical planning sequence (balanced steady-state free-precession, resolution 1.5x1.5x1.5 mm^3^). Spatial integrity was assessed by the Euclidian distance between the control point detected on the image and their theoretical locations. A first plane free of artefact (FPFA) was defined to evaluate the spatial integrity beyond the larger banding artefact.

**Results:**

In the region extending up to 20 mm from the largest banding artefacts, the tested passive and active implants could cause distortions up to 2 mm and 3 mm, respectively. Beyond this region the spatial integrity was recovered and the image could be considered as unaffected by the implants.

**Conclusions:**

We characterized the impact of common implants on a low field MR-LINAC planning sequence. These measurements could support the creation of extra margin while contouring organs at risk and target volumes in the vicinity of implants.

## Highlights

• Metallic implants compromise the spatial integrity of magnetic resonance images.
• Phantom-based geometric distortion analysis with various implants.
• Application in radiotherapy on a 0.35 T magnetic resonance linear accelerator.
• Contouring rules accounting for geometric uncertainties near metallic implants.

## 1 Introduction

Magnetic Resonance Imaging (MRI) has become increasingly integrated into radiotherapy (RT) procedures. It serves not only as an additional modality for enhancing delineation but also as an important tool for target tracking and online adaptive re-planning. However, MR images are potentially subject to geometric distortions [1]. This is particularly relevant as current online adaptive workflows use the MR images of the day as the reference for registration [2], [3]. MR image reconstruction relies on spatial magnetic field variations to encode spatial locations. Those pre-determined spatial variations are corrupted by the presence of metallic implants. The aim of this work is to describe the geometric distortions caused by the most common types of implants on planning images delivered by a 0.35 T MR-linear accelerator (LINAC).

Perturbations of the pre-defined magnetic field distribution are usually grouped into hardware and sample related distortions [4]. Machine dependent distortions include static field (B_0_) inhomogeneities, eddy-current and gradient non-linearity (GNL) [5]. A strategy to assess spatial fidelity relies on the image analysis of a reference geometry as suggested in early works of the AAPM NMR Task group 1 [6]. Phantom measurements based on known geometrical patterns (cylindrical [7], spherical [8] or grid markers [9]) quantify the effects of increased static field (B_0_) inhomogeneities and GNL on the geometry integrity. GNL correction methods have been developed, as they constitute the main source of hardware related distortions [10], [11]. Similar works have been conducted on MR-LINAC systems as the gantry introduces further perturbations to the magnetic field [12], [13].

In parallel, sample-induced distortions include chemical shift and susceptibility artefacts. While the first one accounts for the constant resonance frequency shift of 3.5 part per million (ppm) between fatty acids and water protons, the second depends on the ability of a material to be magnetized in an external magnetic field, i.e. its susceptibility χ [14]. In both cases, the resonance frequency offset increases with B_0_ and results in a signal displacement in the reconstructed image that is proportional to the pixel size (mm) divided by the pixel bandwidth (sequence characteristic, Hz/pixel). Susceptibility artefacts have been well characterized for simple geometries [15] and are expected around 9 ppm at air/tissue interfaces such as the nasal cavity [16]. Tissue induced susceptibility effects are moderate compared to GNL and can be efficiently corrected by active B_0_ shimming and B_0_ mapping [17]. As a result, spatial integrity is well controlled and characterized for MR systems used in RT. The guidelines for planning MR simulation [18] and MR guided radiotherapy (MRgRT) [19] requesting distortions below 1 mm in a 10 cm radius (and accepting up to 2 mm within a 20 cm radius) can be satisfied.

By contrast, metallic implants induce severe susceptibility artefacts. Mitigation strategies aim therefore at making the spatial encoding mechanism more resistant to offset resonances. These include adequate sequence selection [18] and the use of large read-out bandwidth or lower field systems, which are less prone to susceptibility artefacts [20]. Our 0.35 T MR-LINAC employs a balanced steady state free precession (bSSFP) sequence [21] for planning purposes to compensate for SNR loss at low-field. The off-resonance effects are expected to generate banding artefacts (appearing as black fringes) in the bSSFP and to induce spatial distortions confined to the implant vicinity [22]. Distortions caused by metallic implants are strongly system and sequence dependent [23] and not well documented in the context of low field MR-LINAC systems despite the potentially large number of implant wearing patients who could benefit from an online adaptive RT treatment. As an example, a study investigated the feasibility of prostate treatment in the presence of hip prostheses (1.5 T MR-LINAC [24]), another investigates the dosimetry impact of bilateral hip implants on photon treatments [25]. Besides passive implants, a patient with an implantable cardioverter defibrillator (ICD) was treated on the 0.35 T MR- LINAC for ventricular tachycardia [26] underlining the need for characterizations of distortions induced by active implants.

This article does not address safety considerations covered in international standards such as the IEC6061 report or in the ISO10974. In this work, we propose a systematic phantom based measurement of the displacements caused by different types of passive and active implants on our planning images (bSSFP sequence, 0.35 T MR-LINAC) in order to derive a rule for the management of distortions in patients with passive and active metal implants.

## 2 Materials and methods

### 2.1 Spatial integrity phantoms

**Fig. 1:**
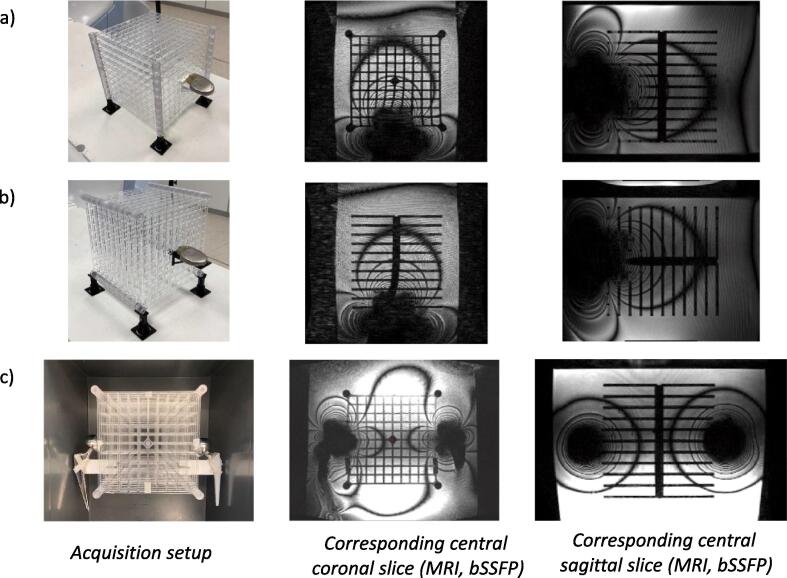
a) Phantom in coronal orientation with an ICD (part reference P1) attached to the middle plane b) Phantom in transversal orientation with an ICD (part reference P1) attached to the center of plane c) Phantom in coronal orientation with femoral stem and head implants attached on the left and right sides (setup parts: 13 + 5 and 12 + 4).

A large field MR image distortion phantom (604-GS, CIRS, Norfolk, USA) was used to characterize our system and benchmark our distortion analysis software (details of the geometry are presented in Supplementary Figure S1.a). However, this phantom did not allow the insertion of any external object such as hip prosthesis. Thus, a custom-made 3D phantom was used to assess the spatial integrity in the presence of medical implants. This phantom covers a field of view of 20x20x20 cm^3^ and is composed of eleven plates, each presenting 116 control points. A control point is defined by a planar cross pattern (3.8 mm branches, spacing 19.6 mm). The phantom is presented in Fig. 1a-b and the details of the geometry is provided in Supplementary Figure S1.b. Additional holders were 3D printed to attach the implants directly to the reference grid. The custom phantom was immersed in a 30 L polypropylene tank (40x30x32.5 cm^3^). The CIRS and custom phantoms were filled with distilled water.

### 2.2 Implants

This study included passive implants used in hip and shoulder arthroplasty procedures such as femoral and humeral stem parts; femoral and glenoid heads; acetabular part, made of different materials: cobalt-chrome (CoCr), titanium (Ti) and stainless steel. In addition, three active implants (two ICDs and one pacemaker) were investigated. Among those, one ICD was not MR conditional.

The implants were attached to the reference grid at the center of the phantom outer plane (single implant setup, Fig. 1a-b). Since our custom phantom is a stack of 2D plates, two acquisitions were performed with the reference patterns covering the coronal and transverse planes, respectively (Fig. 1a-b). The list of investigated configurations is presented in Table 1a.

**Table 1:** Table 1a: Results for the single implant setups, implants are positioned as depicted in Fig. 1a and 1b. The first plan free of artefacts (FPFA) is defined as the closest transversal plane free of banding artefacts. Table 1b: Results for the bilateral setup, implantable systems are positioned as depicted in Fig. 1c. The FPFA are defined as the closest sagittal planes on the left and right sides free of banding artefacts. *The entire field of view is contaminated.

Description	Part reference	Phantom orientation	Mean whole FOV (mm)	p95whole FOV (mm)	Max whole FOV (mm)	FPFA position (mm)
**1.a) Unilateral implant configuration**						
None	none	coronal	0.626	1.075	1.640	0
		transversal	0.690	1.086	1.777	0
Light stainless steel femoral head	4	coronal	0.782	1.363	3.391	100
		transversal	1.108	2.026	3.493	100
Heavy Stainless steel femoral head	5	coronal	0.809	1.442	3.389	100
		transversal	1.049	1.696	2.417	100
carbon distal plate	6	coronal	0.643	1.076	1.612	0
		transversal	0.695	1.082	1.621	0
Ti femoral stem part	7	coronal	0.626	1.057	1.594	20
		transversal	0.679	1.082	2.723	20
Ti humeral stem part	8	coronal	0.630	1.087	1.623	0
		transversal	0.696	1.097	1.650	20
Shoulder cap.	9	coronal	0.626	1.040	1.858	20
		transversal	0.679	1.048	3.819	20
Titanium 1 (femoral head)	10	coronal	0.641	1.066	1.778	20
		transversal	0.682	1.086	1.601	20
Titanium 2 (femoral head)	11	coronal	0.615	1.070	1.504	0
		transversal	0.679	1.068	1.377	20
Total hip	7 + 16	coronal	0.643	1.070	2.694	20
		transversal	0.685	1.078	1.815	20
P1	1	coronal	1.698	3.086	5.805	200*
		transversal	1.465	2.651	5.918	200
P2	2	coronal	1.563	2.718	5.868	200*
		transversal	1.394	2.472	4.740	160
P3	3	coronal	0.784	1.463	5.527	120
		transversal	0.876	1.488	1.934	120

**1.b) Bilateral implant configuration**						
None	none	coronal	0.570	1.165	1.813	0
						0
bilateral femoral heads: stainless steel	5 left	coronal	0.666	1.460	5.785	80
	4 right					0
bilateral femoral stem part: stainless steel + Ti	13 left	coronal	0.590	1.202	5.704	40
	12 right					20
bilateral fem. stem Ti + (Ti and CrCo heads)	7 + 16 left	coronal	0.594	1.169	5.823	20
	12 + 15 right					20
femoral stem Ti + head CoCr	7 + 17 left	coronal	0.623	1.299	4.151	20
femoral stem Ti + head stainless steel	12 + 5 right					80
femoral stem + head stainless steel	13 + 5 left	coronal	0.729	1.617	5.878	100
femoral stem Ti + head stainless steel	12 + 4 right					80

Patients presenting with bilateral hip prosthesis are not rare [27]. To further characterize this situation, a setup with one implant attached on each side of the grid (set in the coronal orientation, Fig. 1c) was also analyzed. The induced artefacts are known to be additive and the setup was used to characterize different elements (femoral stem part, femoral heads and spacer) simultaneously. The list of bilateral implant combinations is reported in Table 1b. A detailed implant description with their positioning is provided in Supplementary Table S8.

### 2.3 Data acquisition

All images were acquired using a clinical planning protocol on a 0.35 T MR-LINAC (MRIdian, ViewRay, inc. Denver, USA). The imaging protocol chosen used a bSSFP sequence (repetition time TR = 3.37 ms, echo time TE = 1.4 ms, FOV = 450x300x360 mm^3^, resolution 1.5x1.5x1.5 mm^3^, bandwidth 538 Hz/pixel) with a slice partial Fourier acquisition (factor 7/8, head-feet direction) and no parallel imaging. The gantry position was fixed at 330° and the shimming was set in “tune-up” mode (i.e. without any setup specific magnetic field optimization) in order to replicate our treatment simulation conditions. The manufacturer offered different variations of the aforementioned bSSFP. However, they all shared a high receiver bandwidth (>500 Hz/pixel) and the image orientation (transverse) which are known to be critical parameters in terms of geometric accuracy [22], [28]. A detailed analysis of the clinical planning protocols is provided in Supplementary Table S6. A pair of torso coils (6 channels, radiation transparent surface coils) was used for all the measurements.

### 2.4 Data evaluation

The spatial integrity was analyzed by an in-house program using a template matching method to detect marker locations. Those locations were compared with the theoretical grid geometry. Spatial integrity measurements using similar phantoms have been described by several groups [12], [29]. Our method implemented the crucial steps reported in those works: Procrustes registration of the theoretical grid [30], sub-volume up-sampling and template similarity measure. The normalized cross correlation (NCC) was used together with a connected component approach to estimate the position of the similarity maximum. A threshold on the NCC was fixed to 0.65 to discard points in regions with banding artefacts or larger air bubbles while preserving distorted regions. This value is in line with previous work (0.75 is used in [12] for images with a 1.5 mm resolution). A detailed description including validation results is presented in the Supplementary Material.

The same method was used to analyze the spatial integrity in the presence of implants.

The Procrustes transformation was estimated in absence of implantable device and applied on the acquisitions with implants to avoid the grid registration being affected by the artefacts. For each setup, we report the distortion mean, maximum and 95th percentile (p95, as a more robust estimate of the maximum) values. To characterize the spatial integrity recovery close to banding artefacts, a first plane free of artefact (FPFA) was defined as a surrogate for the closest uncontaminated region. Artefacts were expected to extend mostly in the B_0_ direction and to cause larger distortions along the frequency encoding direction (left–right in our case) [28]. In the single implant case, planes orthogonal to B_0_ were investigated to replicate the worst-case scenario. For the bilateral setup, FPFA are reported among sagittal planes (Fig. 3c). Its position can be interpreted in the transverse plane as the closest uncontaminated region with respect to our grid spacing of 19.6 mm.

## 3 Results

### 3.1 Spatial integrity with one implant

**Fig. 2:**
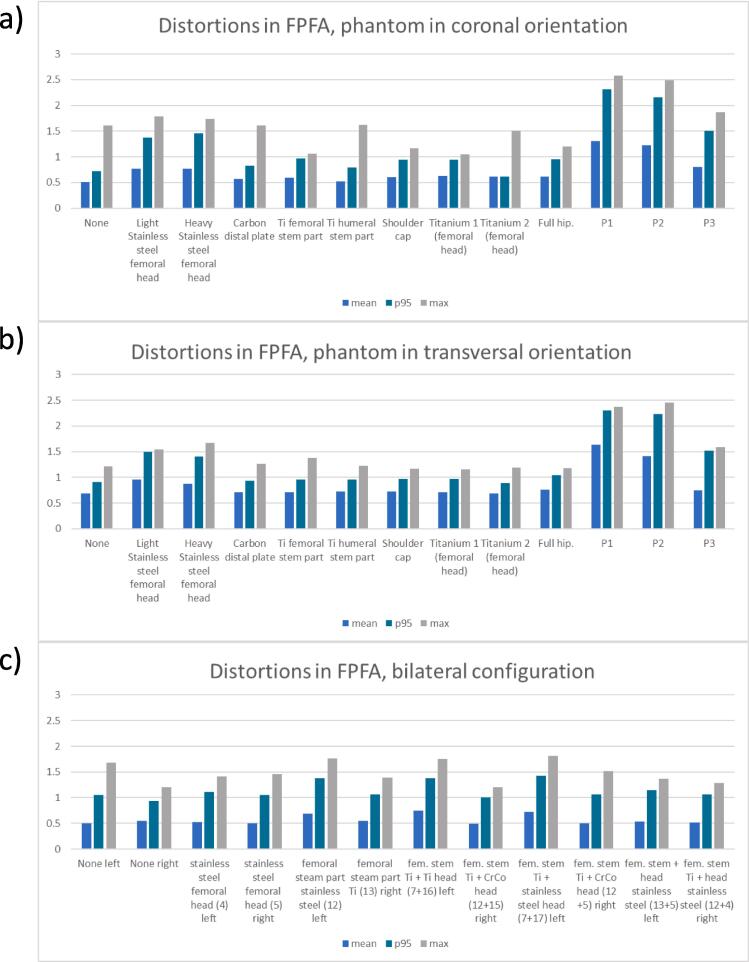
Mean, 95th percentile and maximum distortions in the transverse first plane free of artefacts (FPFA) with the phantom in the coronal orientation (2a) and in the transversal orientation (2b). The results for the bilateral setups are reported for sagittal FPFA with implants on the left and right sides separately in 2c.

**Fig. 3:**
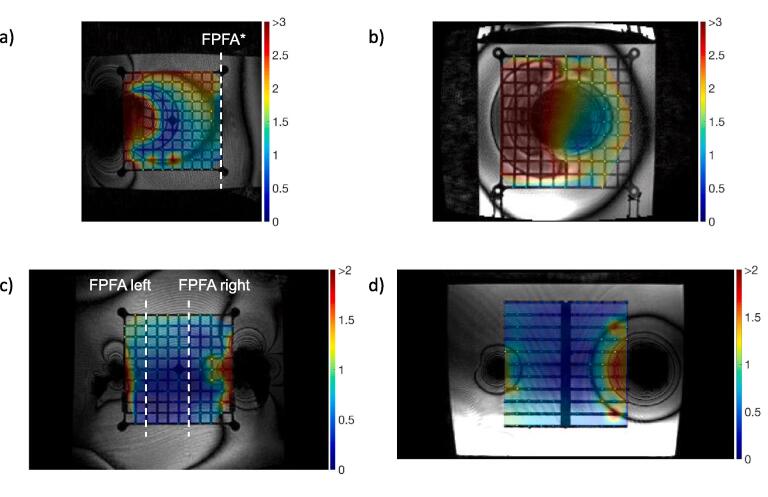
a. 2D-distortion map interpolated from marker displacements, no marker is displayed for outliers. Dark red areas correspond to distortions > 2 mm for passive implants and > 3 mm for active implants a) ICD (part reference P1), *iso*-center coronal view, phantom in the coronal orientation, FPFA* is defined as the last transverse plan as the whole FOV is contaminated b) ICD (part reference P1), transversal view closest to the implant, phantom in the transversal orientation c-d) Setup parts 7 + 17 (Ti + CoCr) on the left, 12 + 5 (Ti + stainless steel) on the right, transversal and coronal views. FPFA are reported for sagittal planes in the bilateral setup. (For interpretation of the references to colour in this figure legend, the reader is referred to the web version of this article.)

**Fig. 4:**
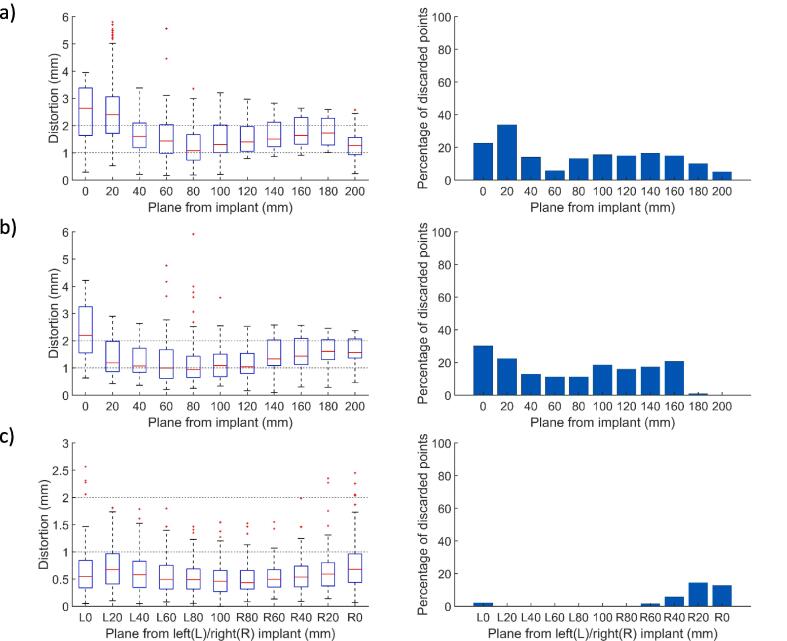
Left part: Distortion in mm per transversal planes (median, 25th, 75th percentile, outliers in red) vs. distance to implant. Right part: percentage of discarded points vs distance to implant. Fig. 4a, 4b correspond to the setups in Fig. 1a and 1b, respectively; 4c corresponds to the setup parts in Fig. 3c and 3d. (For interpretation of the references to colour in this figure legend, the reader is referred to the web version of this article.)

Table 1a summarizes the mean, maximum and p95 distortions (mm) across the custom phantom in both orientations (coronal and transversal) for 12 implantable systems. The mean distortion was found to be below 0.7 mm for the majority of systems with the exception of active devices and stainless-steel implants. The position of the FPFA reflects the extension of the fringes of the artefacts depending on the implant. For titanium pieces, only the plane directly in contact with the implant is affected and the artefact fringe does not extend more than 20 mm away from the implants end in the B_0_ direction. On the contrary, this value increases to 100 mm in the presence of stainless steel pieces. Active devices corrupt the whole field of view (Fig. 1a-b). Beyond the last artefact fringe the mean distortion over passive implants configurations is 0.69 ± 0.11 mm and the p95 1.01 ± 0.24 mm which is in agreement with the vendor constraints. With active implants those values increase to 1.19 ± 0.35 mm and 2.00 ± 0.39 mm, respectively (Fig. 2a-b). The restoration of spatial integrity is visually confirmed in the interpolated distortion maps as proposed in Fig. 3a-b. Fig. 4a and 4b illustrate how the measured distortion per plane is modulated by the fraction of discarded points, the distance to the iso-center (located 100 mm away from the implant) and the phantom orientation.

### 3.2 Spatial integrity with two implants

Table 1b reports the mean and maximum distortions measured on the custom phantom set in coronal orientation with implantable systems attached on both left and right sides. The results are reported for each side separately. The analysis of the FPFA position illustrates the influence of the material on the fringe extension. For every combination of passive implants the vendor constraints were respected in the FPFA (Fig. 2c). The artefacts are greater for stainless steel pieces (up to 100 mm) and smaller for Ti and CoCr implants (20 mm). The bilateral setup also verifies the additive nature of the contamination patterns with the FPFA position determined by the worst material in the assembly. For example, pieces 12 + 5 (Ti + stainless steel) on the right contaminates the image up to 80 mm while the assembly on the left 7 + 17 (Ti + CrCo) degrades the image only up to 20 mm (Fig. 3c-d and 4c).

## 4 Discussion

In this study; we quantified the distortions caused by the presence of common active and passive implants on our bSSFP planning sequence at 0.35 T. We report the position of the first orthogonal phantom plane free of artefacts together with the spatial distortions measured in this plane. We chose a phantom design based on stacked planes to cover a large volume (20x20x20 cm^3^) while benefiting from the in-plane accuracy of the manufacturing tools. The average measured distortions over all control points (maximum distance from the iso-center: 16 cm) without implants were respectively 0.63 mm (maximum 1.64 mm) and 0.69 mm (maximum 1.78 mm) for the phantom planes positioned in the coronal or transversal directions. A 3D grid as implemented in the CIRS 604-GS phantom could yield more accurate results but is difficult to produce with a large grid thickness. The 3D printed design proposed by Jafar et al. [29] uses 2 mm vertices, which are insufficient for a 1.5 mm imaging resolution. The distortions measured using the CIRS 604-GS phantom and its commercial software were on average 0.61 mm within a 16 cm radius (maximum 1.589 mm within the 15.5–16 cm spherical band) for our system. In view of these results, the proposed custom-made phantom together with our template matching method are adapted to measure spatial integrity. Not all low field MR-LINAC versions benefit from the gantry dependent shimming optimization that improves B_0_ homogeneity over gantry positions [31]. In this study, we used our simulation reference gantry angle. A summary of related spatial integrity publications is provided in Table 2 for comparison.

**Table 2:** Summary of related publications reporting hardware and sample induced distortion for different MR based treatment delivery systems.

Publication	Systems	Reported distortions	Comments
Ginn et al. [12]	0.35 T MR- cobalt 60	mean/maximum: 0.37/1.15 mm at 10 cm radius 0.49/1.88 mm at 17.5 cm radius	Hardware related distortions
Nejad-Davarani et al. [40]	0.35 T MR-LINAC	100 % of the points with less than 1 mm distortion in the 10 cm radius and 96 % above 2 mm within the 20 cm radius.	Hardware related distortions
Marasini et al. [41]	0.35 T MR-LINAC	Distortion reported in an extended field of view (mean 0.8 mm in the 300–400 mm region).	Hardware related distortion assessed with different phantoms.
Marasini et al. [42]	0.35 T MR-LINAC	The mean distortion in the central transverse plan can be reduced from 0.33 to 0.18 mm using a deformation vector field determined using the quasar MRID phantom.	This work demonstrate how geometry accuracy can be further improved by using separately-measured deformation vector fields.
Lewis et al. [43]	0.35 T MR-LINAC, 1.5 T and 3 T diagnostic systems	Simple length measurements on the ACR and Insight phantom at different field strength with similar results. The authors note a lower spatial resolution at low field.	Field strength influence on various imaging quality parameters including geometry accuracy.
Roberts et al. [44]	1.5 T MR-LINAC	a multi-institutional study reports a maximum (99th percentile) displacement of 0.7 mm within a 7.5 cm radius and 2 mm within a 17.5 cm radius	Hardware related distortions in a multi-institutional study using the vendor QA tool based on a spin echo (SE) sequence
Stanescu et al. [45]	1.5 T MR-LINAC	Composit distortion in the liver of mean 0.4, maximum 1.4 mm in a cohort of 16 patients	Hardware related distortions measured with a Quasar MRID phantom and sample related distortion estimated from susceptibility maps and finite difference method
Neylon et al. [46]	0.35 T MR-LINAC	Maximum mean distortion of 2.01 mm at the bone/tissue interface using landmarks on CT and MRI	Sample induced distortion at different material interfaces in a 10 patient cohort.

Standards to estimate artefact contamination from passive implants such as the ASTM F2119 recommend the use of a paramagnetic solution (CuSO_4_, NiCl_2_) to shorten T1 and reduce scan time. In our experiment, distilled water yields good contrast. In addition, dielectric effects that might occur while imaging large water tanks are not a concern at 0.35 T [32]. Similarly, with a relative susceptibility to water of −0.03 ppm, the use of acrylic prevent any further susceptibility artefacts originating from the phantom itself.

Metal artefacts depend on numerous factors such as material, shape and orientation with respect to B_0_, design (active vs passive) and field strength. Therefore, the reported results cannot be transferred to higher field strengths. Koff et al. [33] evaluated the influence of cylindrical samples made of stainless-steel, CoCr, Ti and ultra-high molecular weight polyethylene on a reference geometry. Another method analyses the registration of reference grids to an artefacts free MR scan [34]. Those studies focus on a 2D turbo spin echo revealing different artefact patterns but conceptualize the presence of 3 regions: non-recoverable area, distorted but recoverable and unaffected area. We use a similar strategy by distinguishing planes with and without banding artefacts, as we are mostly interested in the unaffected area for RT planning. In a clinical context, susceptibility artefacts caused by hip prosthesis can be assessed by B_0_ map techniques. Our system does not enable B_0_ mapping sequence in clinical mode and it was therefore not investigated in this work. Moreover the problem of phase unwrapping introduces additional uncertainties in distortion quantification [24], an inevitable trade-off when no reference geometry is available as in in-vivo cases.

The distance of the FPFA from the implant (Table 1) reflects the expected influence of the material for passive implants. The larger contaminations are reported for stainless steel followed by CoCr and Ti. The pattern of contamination is complex, however the average and maximum distortions in the FPFA yield a concise metric to assess spatial integrity recovery. This is confirmed by the visual analysis of the distortion maps in Fig. 3. Our grid spacing (19.6 mm) is a limiting factor, therefore only the area located 20 mm beyond the last fringe can be safely considered as unaffected by the implant. The Fig. 4 reports isolated maximum distortions up to 6 mm for the worst cases (active implants Fig. 4a-b and stainless steel Fig. 4c) only in the vicinity of the implants. In the intermediate area separating the last artefact fringe and the closest control point of the FPFA, the p95 over the whole FOV (Table 1) can be considered as a reasonable distortion upper bound and remains below 2 mm. In the presence of active implants, 3 mm is a safer value. According to Scheffler et al. [35], the bSSFP sequence tolerates off-resonance effects up to approximately ± 3π/4TR before signal loss that corresponds to a ± 0.62 mm distortion for our sequence. This further supports 2 mm as a reasonable upper bound assuming that B_0_ homogeneity is restored beyond the last black fringe. These measurements can further support the creation of extra margins while contouring OARs and target volumes near the implants. Patients with hip implants presenting at our institution for prostate cancer treatment are not rare considering the percentage of the Swiss population undergoing hip arthroplasty after the age of 60 (>20 %) [36]. Van Lier investigated the impact on contours of unilateral hip implants by the mean of B_0_ maps and did not report any variations bigger than 1 mm except for the skin contour (1.7 mm) [27]. One of the main limitations of this study is the inability to cover all types of implants. The reported results could also potentially be improved by using active shimming which is currently only available in MR offline mode and therefore not applicable during patient MR simulation. In addition, sequences reducing metal artefacts available on diagnostic MR systems (Slice Encoding for Metal Artifact Correction SEMAC, multiacquisition variable-resonance image combination MAVRIC) [22] could possibly be ported on low-field MR-LINACs. A variation of the bSSFP sequence has been proposed to reduce metal artefacts and could potentially enlarge the unaffected area [37] at the cost of extra acquisition time. Further work could investigate the impact on the real-time CINE images used for gating. Recently an AI-based geometric correction method has also been proposed [38] as well as a real-time geometric distortion correction based on phantom measurements [39], however the diversity of implantable devices might restrain this type of correction strategy to routine cases.

We proposed a method to describe the geometric distortions caused by common types of implants on planning images delivered by a 0.35 T MR-LINAC. We conclude that the region located 20 mm beyond the largest banding artefact can be considered as unaffected by the tested passive implants while the closer region might experience geometric distortion up to 2 mm. The active implants considered in this study further compromised spatial integrity and exhibited deviations up to 3 mm in this 20 mm region. We believe that these measurements could support the creation of an extra margin while contouring OARs and target volumes in the vicinity of implants. From an imaging point of view, implants should not be a systematic exclusion criteria for low field MR-LINAC treatments as long as the unaffected image area enables safe contouring.

## CRediT authorship contribution statement

**Bertrand Pouymayou:** Conceptualization, Supervision, Investigation, Software, Visualization. **Yoel Perez-Haas:** Investigation, Software, Visualization. **Florin Allemann:** Resources. **Ardan M. Saguner:** Resources. **Nicolaus Andratschke:** Resources, Writing – review & editing. **Matthias Guckenberger:** Resources, Writing – review & editing. **Stephanie Tanadini-Lang:** Conceptualization, Supervision, Writing – review & editing. **Lotte Wilke:** Conceptualization, Supervision, Writing – review & editing.

## Declaration of Competing Interest

The authors declare the following financial interests/personal relationships which may be considered as potential competing interests: M. Saguner: AMS received speaker/advisory board/consulting fees from Bayer Healthcare, Biotronik, Daiichi-Sankyo, Medtronic, Novartis, Pfizer and Stride Bio Inc. B. Pouymayou and N. Andratschke have previously received compensation by ViewRay, Inc. for consulting work.
