# Supplementary material for: Characterization of spatial integrity with active and passive implants in a low-field magnetic resonance linear accelerator scanner
Source: Phys Imaging Radiat Oncol. 2024 Apr 7;30:100576. doi: 10.1016/j.phro.2024.100576 (PMC11031795; doi:10.1016/j.phro.2024.100576)
Supplement: Supplementary Data 1 [file mmc1.docx]

**Supplementary Material**

**Marker detection method and validation**

1. **Phantom description**

A large field MR image distortion phantom (604-GS, CIRS, Norfolk, USA) was used to characterize our system and benchmark our distortion analysis software. This phantom presents 2152 control points defined by the vertices of 3D crosses (Supplementary Figure S1.a, 3 mm diameters rods, 20 mm spacing). The markers spatial arrangement is illustrated in Supplementary Figure S2.c.

A custom-made 3D phantom was used to assess the spatial integrity in the presence of medical implants. This phantom was made of 11 parallel acrylic plates (PMMA, 5 mm thickness, spaced by 20 mm, Supplementary Figure S1.b) and covers a field of view (FOV) of 200x200x200 mm^3^. Each plate was cut to form a 11x11 grid pattern (3.8 mm thickness, 19.6 mm spacing) defining 116 control points (the 4 corners and the middle point were discarded to support the structure as shown in Supplementary Figure S1.b).

**Supplementary Figure S1.** a) 3D cross defining on of the 2152 markers in the 604-GS CIRS phantom b) Custom made phantom of 11 stacked plates of 5 mm (planes spacing 20 mm, in-plane spacing 19.6 mm, 3.8 mm thickness)

| a) | 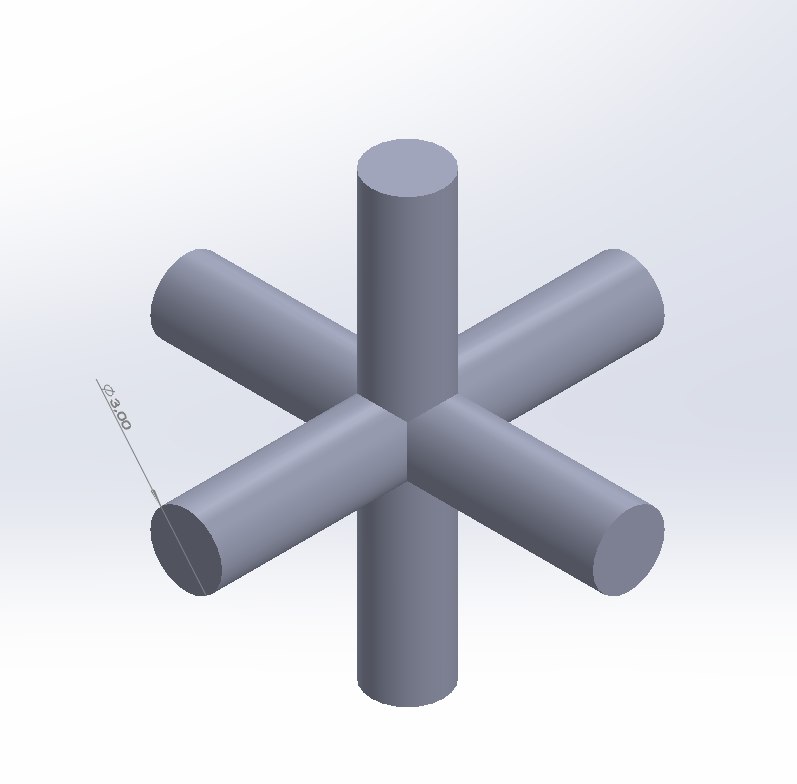 | b) | 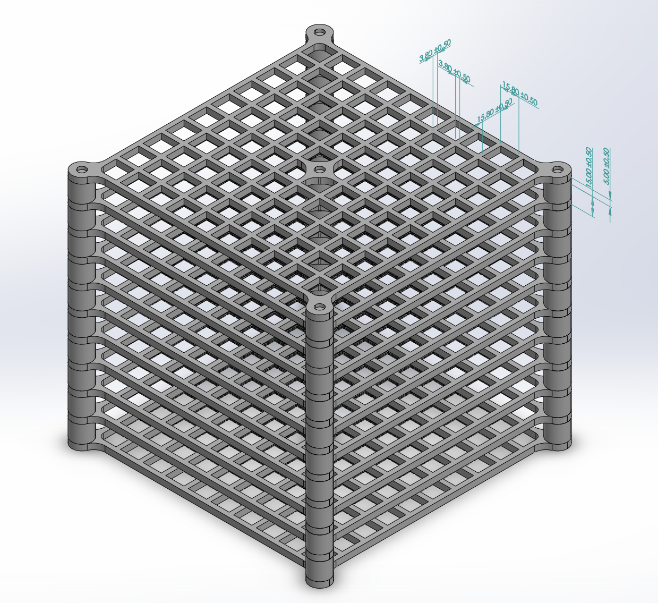 |
| --- | --- | --- | --- |

1. **Method description**

The image distortion is evaluated by comparing the marker location on the image with the expected marker position from the theoretical grid design. The marker detection method employs a template matching technique similar to the one applied by Ginn et al. [1] or Jafar et al. [2] and has been implemented using MATLAB (MathWorks©, R2018a). The normalized cross correlation (NCC) was used as a measure of similarity to detect the marker template within the image. The marker position thus corresponds to the maximum of the NCC function (Supplementary Figure S2.b). The NCC maximum is defined as the center of mass of the pixels exceeding a certain threshold. To avoid multiple maxima, only the center of mass of the largest convex hull was considered. The NCC was computed in a sub volume (size of one reference pattern unit) centered on the expected marker location to reduce computation time (Supplementary Figure S2.b). To improve the localization accuracy the sub volume was oversampled by a factor of three using cubic interpolation. The ability to detect the reference markers depends strongly on the NCC threshold and the size of the template used. These two parameters have been empirically optimized to reject markers located in corrupted regions (banding artefacts, phantom edges) and to detect large distortion (Supplementary Figure S2).

As suggested in Ginn et al. [1] and others [3], the Procrustes method [4] was employed to correct for phantom misalignments. Only the markers located within a 40 mm radius (corresponding to 2 reference pattern units) were considered to determine the phantom displacement and rotation. The estimated transformation was then applied to the list containing all the expected marker locations. The overall distortion figure is obtained by taking the Euclidian distance between the position of the detected markers and the registered theoretical grid. (Supplementary Figure S2.c).

1. **Validation**

Three tests were implemented using the CIRS 604 phantom to assess the performance of our marker detection method implementation. Firstly, we compared the distortion results against the analysis tool provided by CIRS (tools.cirsinc.com). The marker localization performance depends strongly the NCC threshold and the size of the template used. We adjusted those parameters to match the CIRS analysis tool. The results are reported in Supplementary Figure S3; the mean difference over all thin spheres (differential radius 5 mm, 44 spheres in total) is 0.09 mm (+/- 0.08 mm).

Then the marker detection method was used on a CT scan phantom (0.78x0.78x0.6 mm^3^) to evaluate the phantom manufacturing accuracy. The results are reported in Supplementary Figure S4. The maximum distortion is 0.94 mm and the mean distortion is 0.24 mm, which is in line with manufacturer mechanical accuracy specification (1 mm over the whole phantom).

Finally, the custom software was tested on a MR acquisition where the 3D distortion correction was disabled to verify the ability to detect large distortion as illustrated in Supplementary Figure S2.a. The maximum distortion was 7.46 mm and a threshold of 10 mm was applied (half of the grid size) to discard outliers.

**Supplementary Figure S2.** Marker detection method: a) the red box defines the sub-volume selected for the NCC operation b) the NCC function over the sub-volume is represented together with its maximum (red dot) and the corresponding location on the image. The 3D plot shows the applied template and the plane intersection of the maximum

c) Representation of the spatial distortion defined by the Euclidian distance between the sub-volume center and the detected marker

| a)  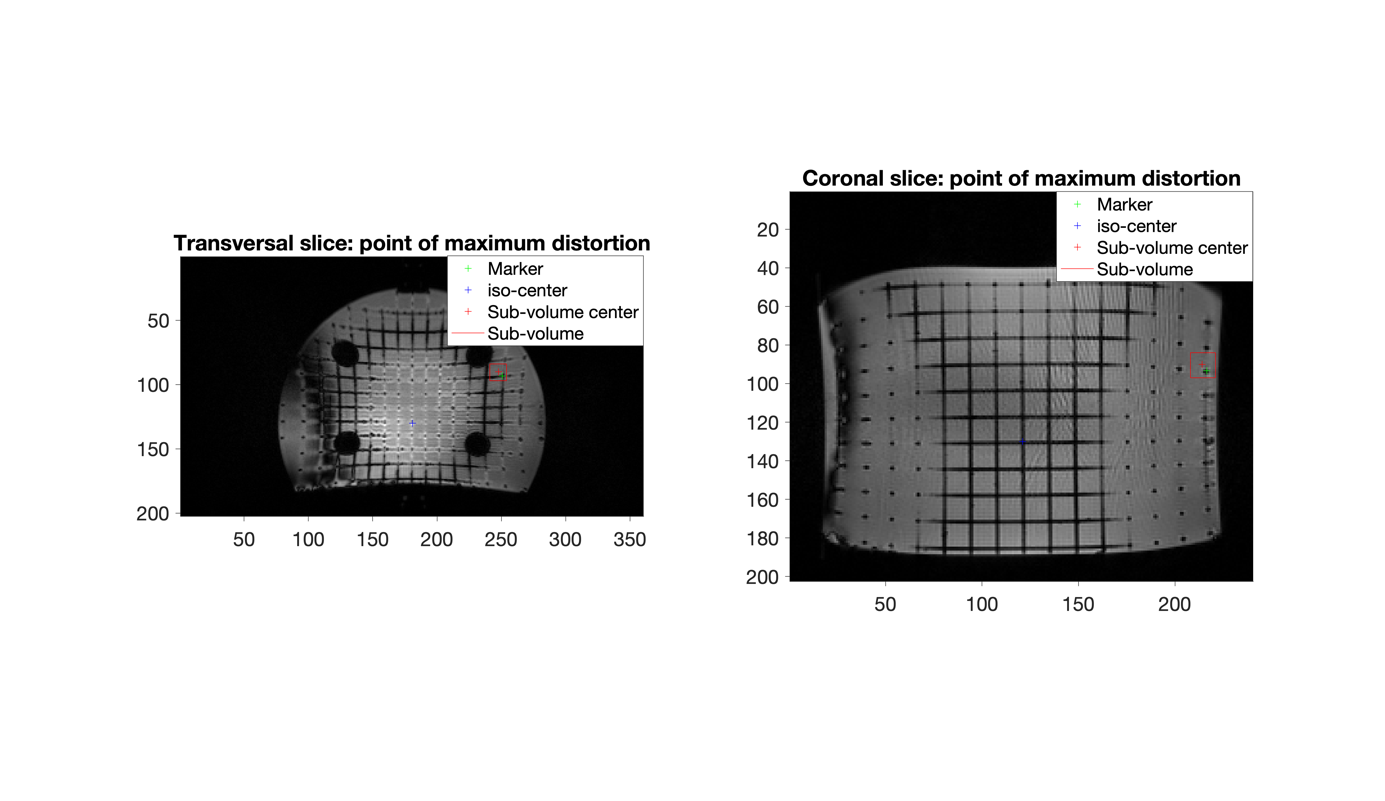  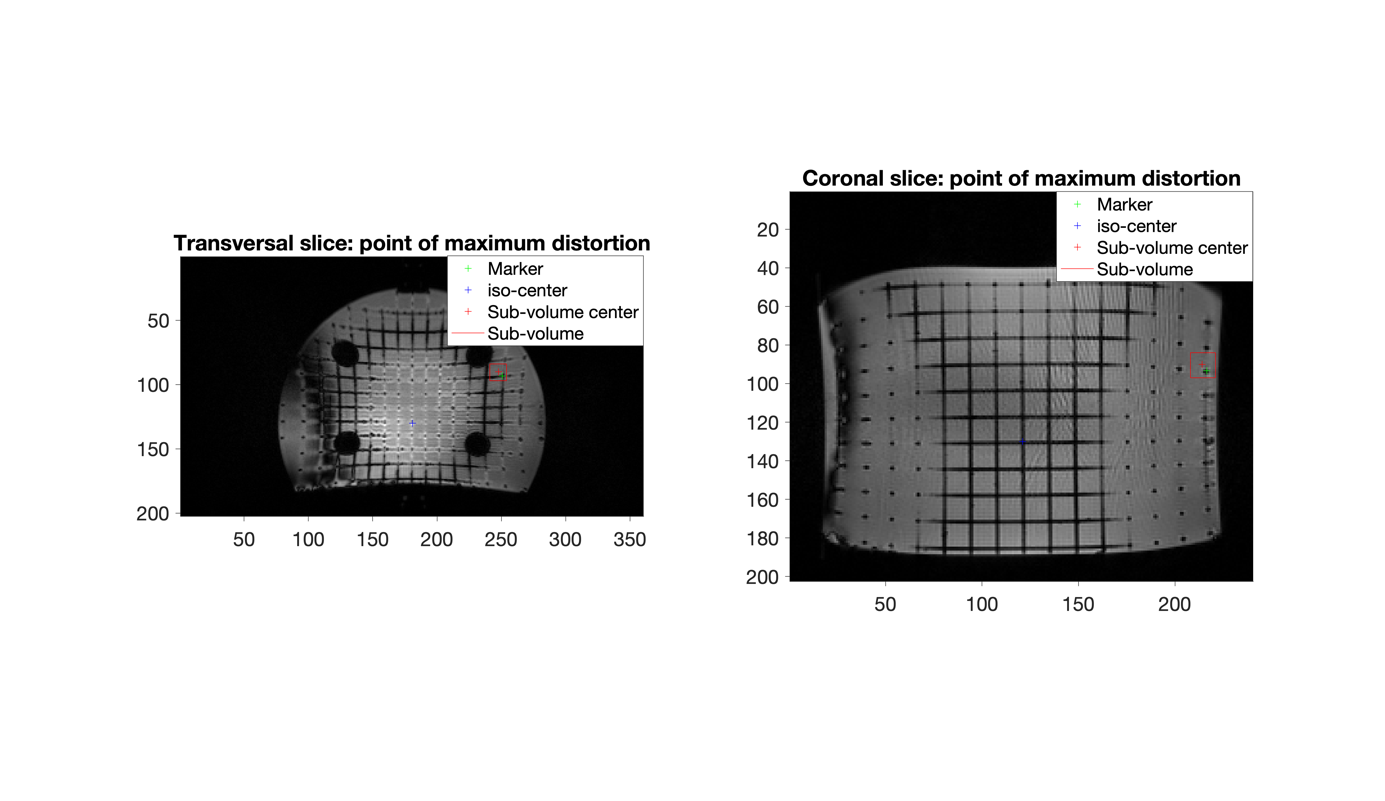 | b)  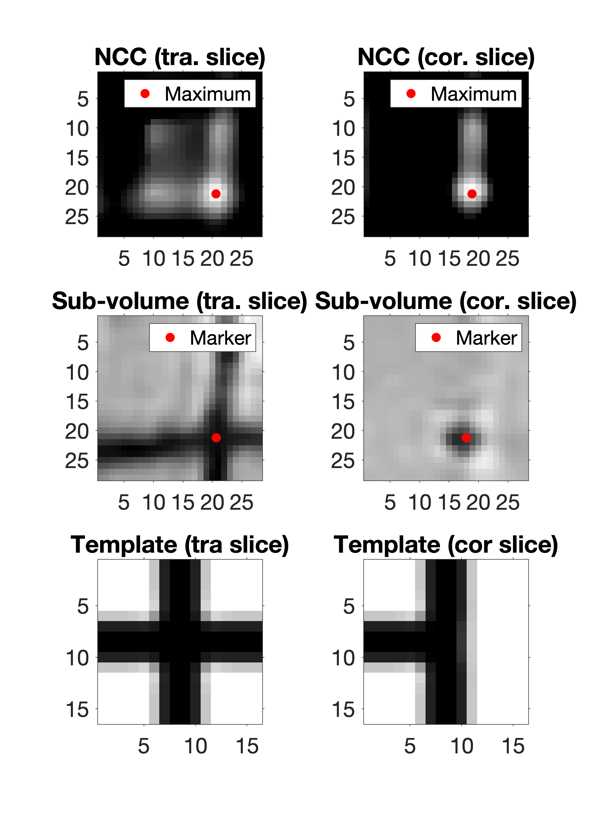  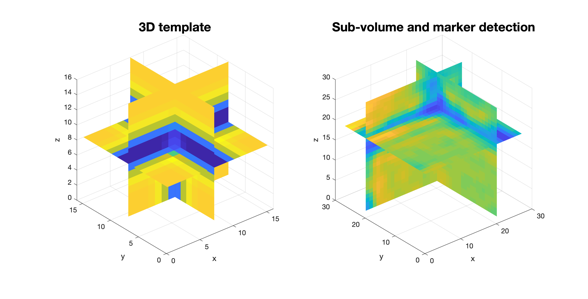 |
| --- | --- |
| c)  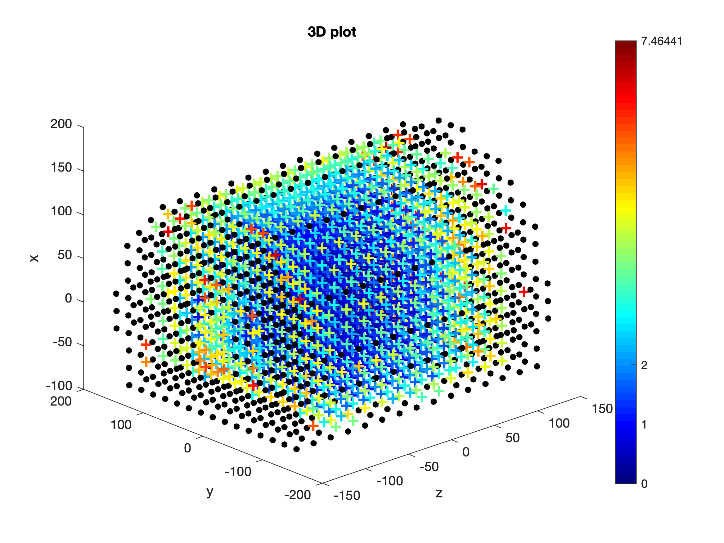 | |

**Supplementary Figure S3.** Average distortion measured in the sphere of radius [r, r+ 5mm] from the iso-center. The blue dotes correspond to the CIRS evaluation software; the red crosses correspond to our custom method. The dashed line represents the vendor tolerance.


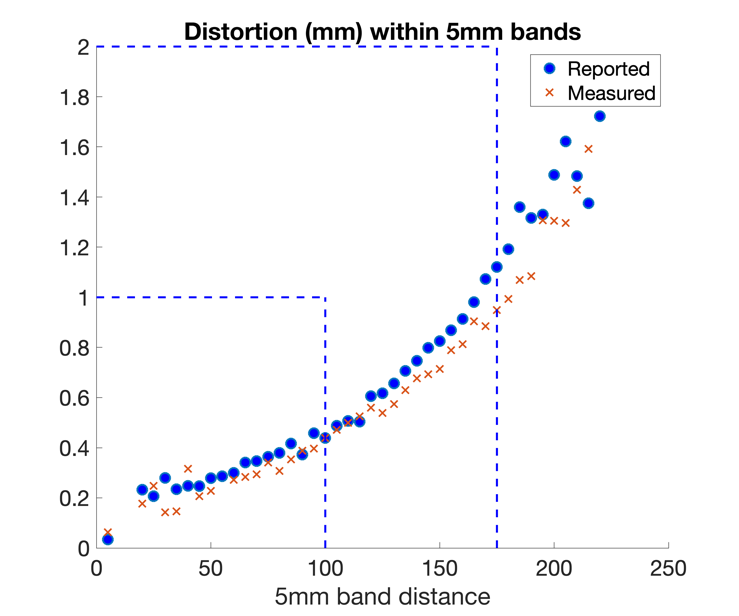


1. **System characterization**

Geometrical distortions are closely related to magnetic field homogeneities. The CIRS 604 phantom was also used to characterize our system and especially the dependence to gantry angles (GA) and shifts from the iso-center. The distortions were measured for gantry angles varying from 0 to 330° with 30° steps. The results are reported in Supplementary Figure S4 with respect to 330° our default imaging gantry angle. The vendor constraints are well respected (<1 mm, 10cm radius, <2 mm, 17.5 cm radius) for all gantry angles; however the imaging iso-center (as determined by the Procrustes registration translation vector) exhibits a maximum variation of 0.85 mm in the transverse plan with respect to the reference gantry position. Lewis et al. [5] reported a comparable maximum iso-center shift of 1 mm across gantry angles. The effect of phantom shifts were also investigated. The phantom was first positioned towards the imaging-isocenter and the couch was then shifted 55 mm in the 6 radiological directions. The band analysis shows minimal deviation to the iso-center position case and all measurements are in agreements with the vendor constraints. The results are reported in Supplementary Figure S5 together with the CT analysis and the scan without 3D distortion correction for comparison.

**Supplementary Figure S4.** Measured distortion in mm vs Gantry angle. The box plot displays the median value (red bar), the 25^th^ and 75^th^ quantile extremities (blue box) and the bar extend to cover 99.3% of the normally distributed data, while red crosses represent points outside of the range. a) Distortion within a 10 cm radius sphere around the MR iso-center b) Distortion within a 17.5 cm radius sphere around the MR iso-center c) Registration shift in the three directions (x=vertical, y=lateral, z=axial) with respect to the imaging iso-center

| a) | 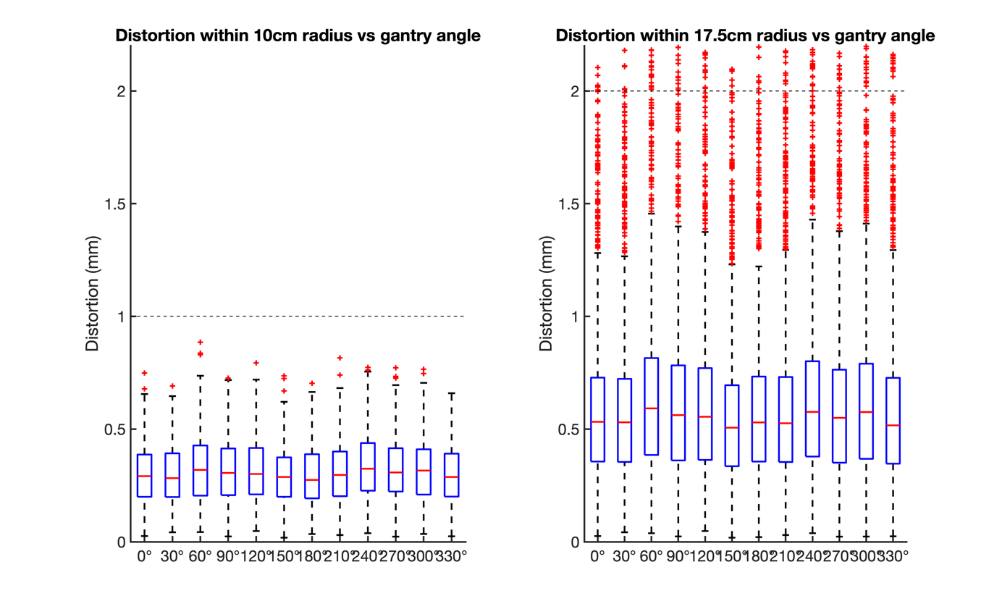 | b) | 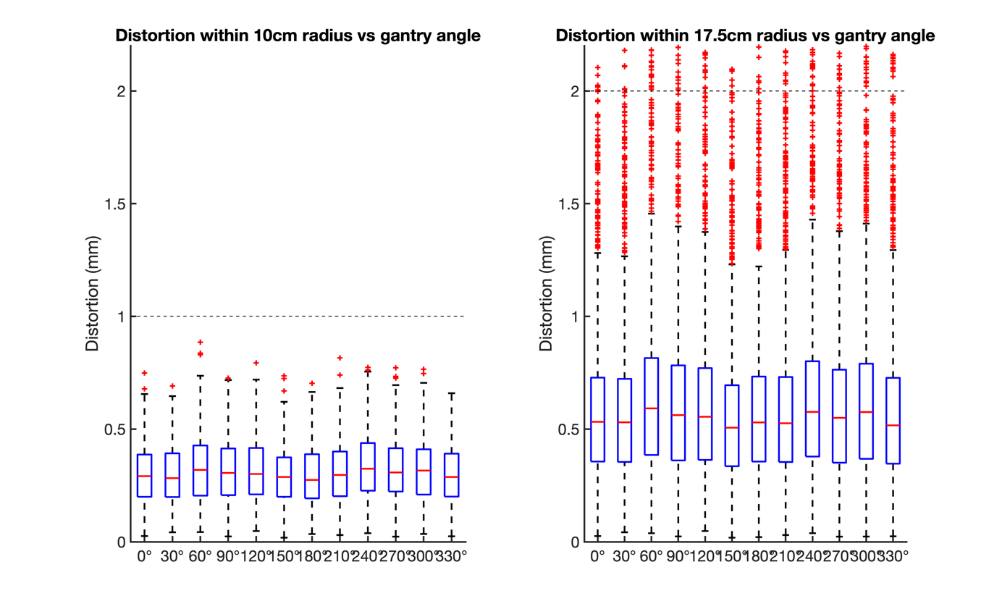 |
| --- | --- | --- | --- |
| c) | 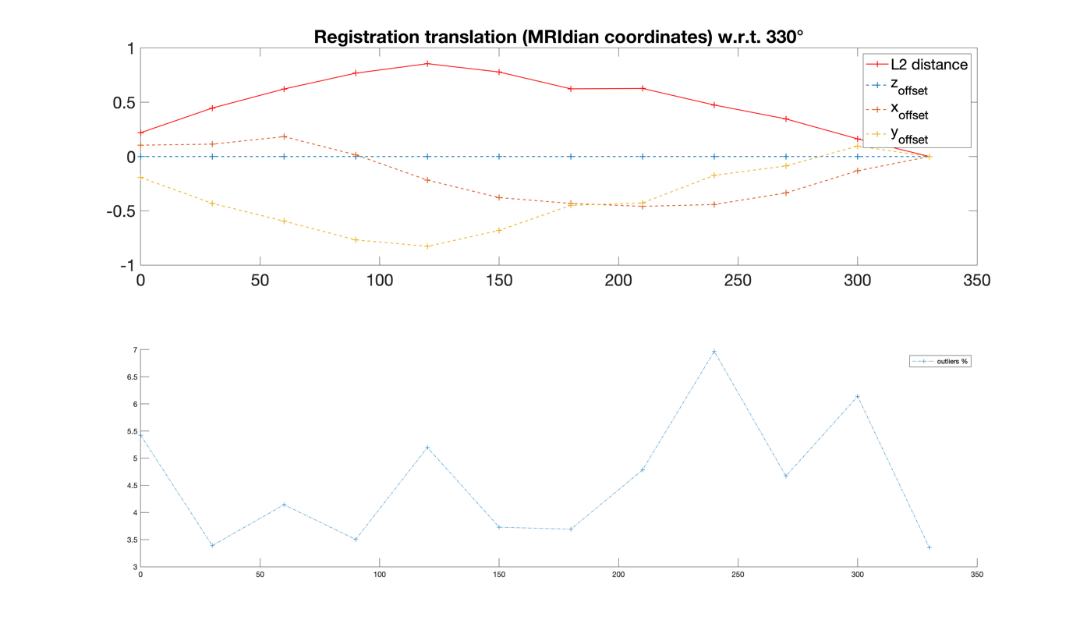 | | |

**Supplementary Figure S5.** Average distortion measured in the sphere of radius [r, r+5 mm] from the iso-center. The orange dots correspond to the iso-center position (reference position) and the blue dots correspond to the evaluation of a CT-scan of the phantom with the same methods. The dark red crosses correspond to the distortion when the vendor distortion correction is turned off. Crosses in the other colors correspond to shifts of 55 mm in the 6 radiological directions.


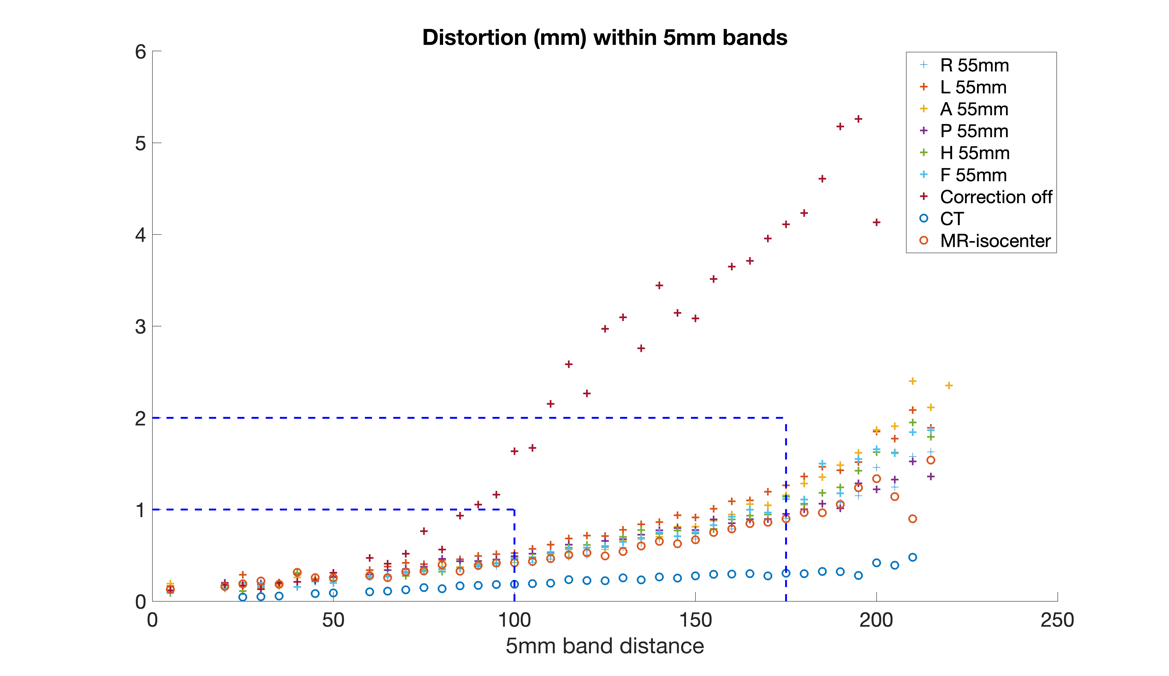


1. **Sequence comparison**

The treatment delivery system (TDS) on the MR-LINAC scanner relies on a unique bSSFP (Siemens, Truefisp) sequence with pre-defined sequence parameters. There are 20 pre-defined protocols varying: the field of view from 400x228x264 mm^3^ to 540x300x360 mm^3^, the acquisition time from 15 s to 175 s, the in-plane resolution from 1.5x1.5 mm^2^ to 1.6x1.6 mm^2^ pixel size and the slice thickness from 1.5 to 3 mm. Note that there is also a GRE sequence available in treatment mode but its long acquisition time (12 min 6 s) makes it impractical in the clinical routine. In terms of spatial integrity parameters such as the bandwidth per pixel, the read-out direction and acceleration methods are expected to play the most significant role. After reviewing the available protocols, we selected 4 sequences covering our phantom field of view and using different acceleration techniques or read out direction: a short sequence using a GRAPPA x2 and partial Fourrier (pF) fractions of 6/8 for both phase and slice sampling. All the sequences were 3D sequences with slab selection, key parameters are summarized in SupplementaryTable S6.

**Supplementary Table S6.** Representative planning protocols on the low field MR-LINAC (bSSFP)

| Sequence short name | Time(s) | orientation | Resolution (mm) | FOV (mm)  RL-AP-HF | Read-out  direction | Bandwith/px | acceleration |
| --- | --- | --- | --- | --- | --- | --- | --- |
| THO | 25 | TRA | 1.5x1.5x3 | 400x400x432 | Right-Left | 537 | GRAPPA x2, pF Slice/phase 6/8 |
| TRA ABD | 172 | TRA | 1.5x1.5x1.5 | 500x449x432 | Right-Left | 535 | pF slice 5/8 pF phase 6/8 |
| COR ABD | 92 | COR | 1.5x1.5x1.5 | 349x360x400 | Head-Feet | 537 | pF slice 6/8 pF phase 6/ |
| TRA HN | 175 | TRA | 1.5x1.5x1.5 | 540x300x360 | Right-Left | 534 | pF slice 7/8 |

**Supplementary Figure S7.** Measured distortion in mm for different planning protocols at GA=330° (see Supplementary Table S6). The box plot displays the median value (red bar), the 25^th^ and 75^th^ quantile extremities (blue box) and the bar extend to cover 99.3% of the normally distributed data, red crosses represent points outside of the range. a) Distortion within a 10 cm radius sphere around the MR iso-center b) Distortion within a 17.5 cm radius sphere around the MR iso-center


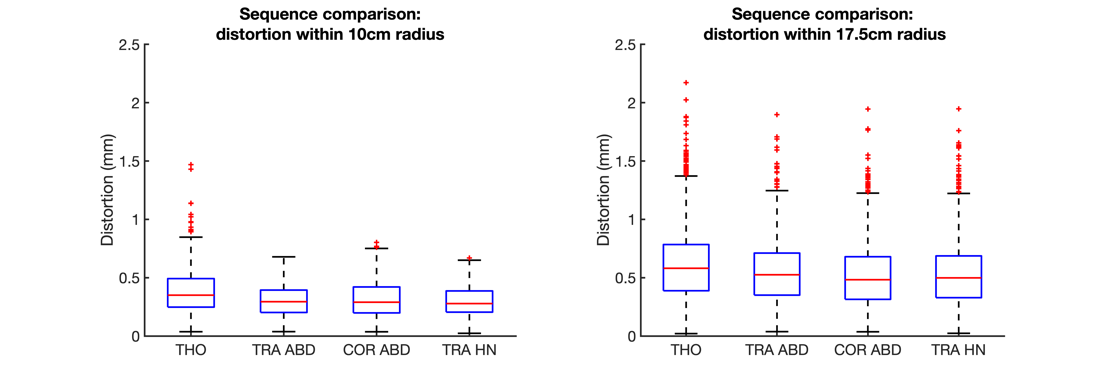


The four sequences performed equally well in terms of average spatial integrity (Supplementary Figure S7). SNR, resolutions and FOV were not taken into account here. The shortest sequence (THO) presented more outliers and presented visually the worst SNR. All the reported values were within the vendor constraints. Since acceleration techniques did not substantially affect the results, we used the sequence (TRA ABD) covering the largest FOV for this study.

**References**

[1] Ginn JS, Agazaryan N, Cao M, Baharom U, Low DA, Yang Y, et al. Characterization of spatial distortion in a 0.35 T MRI-guided radiotherapy system. Phys Med Biol 2017;62:4525–40. https://doi.org/10.1088/1361-6560/aa6e1a.

[2] Jafar M, Jafar YM, Dean C, Miquel ME. Assessment of geometric distortion in six clinical scanners using a 3D-printed grid phantom. J Imaging 2017;3. https://doi.org/10.3390/jimaging3030028.

[3] Huang K, Huang K, Cao Y, Baharom U, Balter JM. Phantom-based characterization of distortion on a magnetic resonance imaging simulator for radiation oncology. Phys Med Biol 2016;61. https://doi.org/10.1088/0031-9155/61/2/774.

[4] Goodall C. Procrustes Methods in the Statistical Analysis of Shape. Journal of the Royal Statistical Society: Series B (Methodological) 1991;53:285–321. https://doi.org/10.1111/j.2517-6161.1991.tb01825.x.

[5] Lewis BC, Shin J, Quinn B, Barberi E, Sievert D, Kim JS, et al. First clinical experience of correcting phantom-based image distortion related to gantry position on a 0.35T MR-Linac. J Appl Clin Med Phys 2021;22:21–8. https://doi.org/10.1002/acm2.13404.

**Supplementary Table S8.** Implants Nomenclature

| **Piece abbreviation** | **Vendor reference** | **Manufacturer** | **Material** | **MR conditional** | **Description** | **Pictures of implant** | **Experimental unilateral setup coronal view** |
| --- | --- | --- | --- | --- | --- | --- | --- |
| 1-P1 | Viva XT CRT-D | Medtronic | Not in the documentation | No | ICD (without electrodes) | 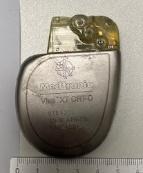 | 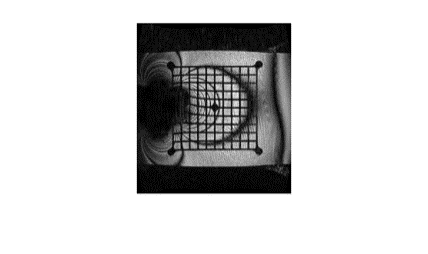 |
| 2-P2 | Clara MRI CRT-D Surescan | Medtronic | Not in the documentation | Conditional 1.5 T,3 T  Max gradient 20 T/m  Max slew rate 200 T/M/s  Normal operating mode | ICD (without electrodes) | 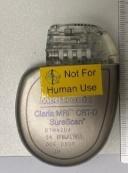 | 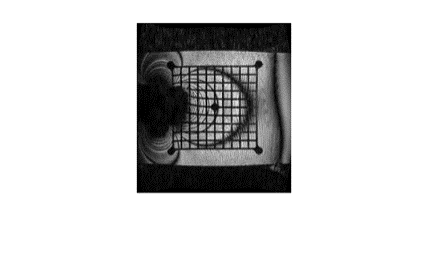 |
| 3-P3 | Essentio MRI (L111) | Boston Scientific | Not in the documentation | Conditional 1.5 T,3 T  First level operating mode | Pacemaker  (without electrodes) | 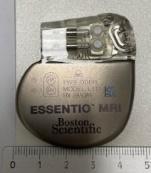 | 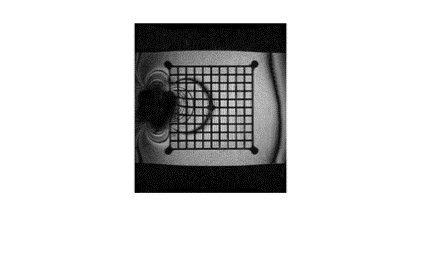 |
| 4-C1 (light) | 2.30.425 | Mathys | FeCrNiMnMoNbN (stainless steel) | No | Ø48 mm. Hemiprothesis femoral head | 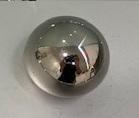 | 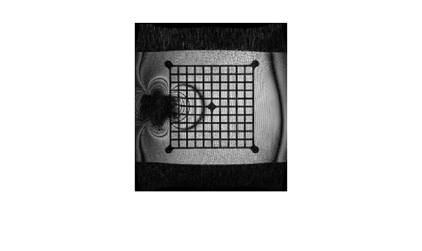 |
| 5-C2 (heavy) | 1.25.152M | Medacta | Nitrogen, stainless steel | Not evaluated in MR environment by the manufacturer | Ø 52 mm. femoral head | 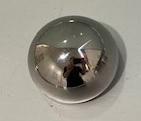 | 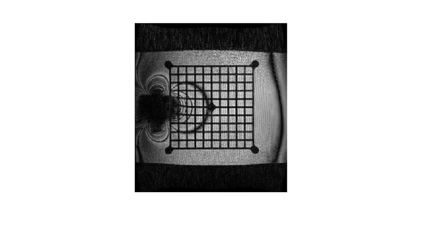 |
| 6-Carb | 22-11-1205 12/01 | Icotec | Carbon | Yes | Distal plate 2 | 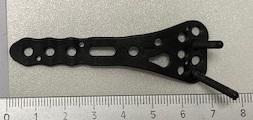 | 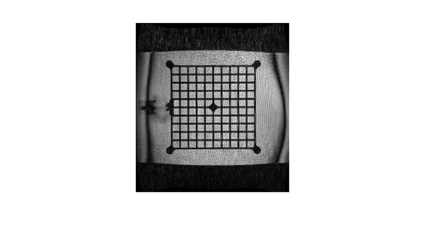 |
| 7-hip | 01.06010.001 / 4019589 12/14 | Zimmer biomet | Titanium alloy | no movement or deflectionexpected at 1.5 T, 3 T, no information about heating | Femoral stem part | 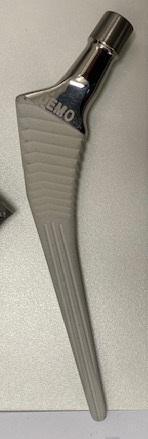 | 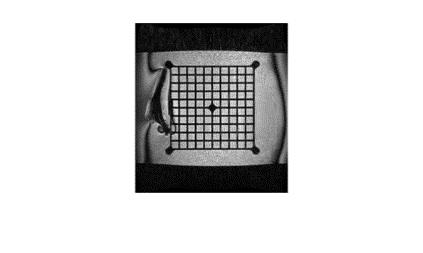 |
| 8-shoulder | 01.04201.072 / 2653177 | Zimmer biomet | Titanium alloy | no movement or deflectionexpected at 1.5 T, 3 T, no information about heating | Humeral stem part | 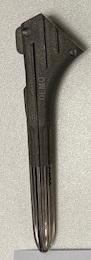 | 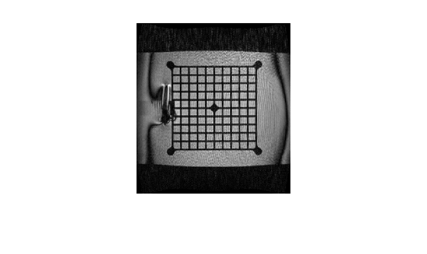 |
| 9-shoulder cup | 01.04223.120 / 2649214 20 retro | Zimmer biomet | Titanium alloy | no movement or deflectionexpected at 1.5 T, 3 T, no information about heating | Humeral cup | 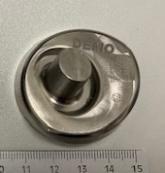 | 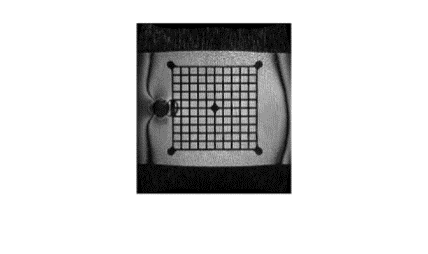 |
| 10-T1 | 01.26.45.0056 | Medacta | Titanium alloy | Not evaluated in MR environment by the manufacturer | Versafitcup acetabular shell | 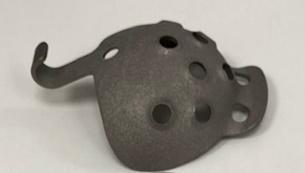 | 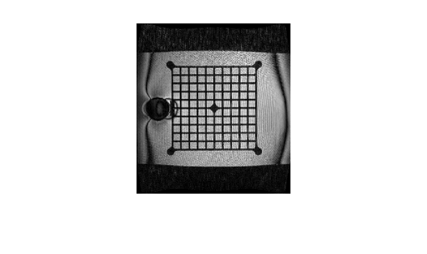 |
| 11-T2 | 94.16.23.62 | Zimmer biomet | Titanium alloy | no movement or deflectionexpected at 1.5 T, 3 T, no information about heating | Acetabular reinforcement ring | 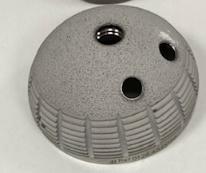 | 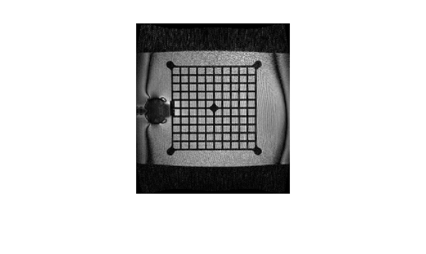 |
| 12-large HIP | 01.12.022 12/14 5°42 | Medacta | Titanium-Niobium alloy with hydroxyapatite coating | Not evaluated in MR environment by the manufacturer | Femoral stem part , AH Coated stems (Quadra-H) | 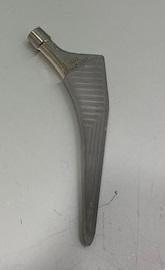 | N/A |
| 13-very large Hip | 01.12.045 12/14 5°42'30 | Medacta | Cemented stem in high nitrogen stainless steel | Not evaluated in MR environment by the manufacturer | Femoral stem part , Quadra®-C | 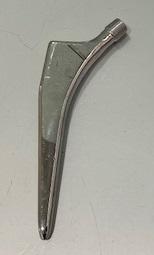 | N/A |
| 14-shoulder head | 01.04212.400 / 2646782 | zimmer biomet | Head is made of  Cobalt Chrome (CoCr28Mo6-Protasul-21WF alloy). | no movement or deflectionexpected at 1.5 T, 3 T, no information about heating | Humeral head implant (anatomical shoulder removal head) | 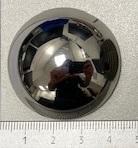 | N/A |
| 15-Femoral head | PLUS16052 28 12/14M | Medacta | CoCrMo | Not evaluated in MR environment by the manufacturer | Femoral head with spacer | 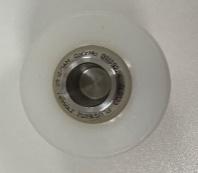 | N/A |
| 16-Femoral cup | 2734784 / 4263 /48 GG | Zimmer biomet | Titanium | no movement or deflectionexpected at 1.5 T, 3T, no information about heating | Allofit acetabular cup | 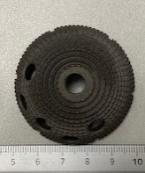 | N/A |
| 17-femoral head | 01.25.011 | Medacta | CoCr | Not evaluated in MR environment by the manufacturer | Femoral head Ø 28 | 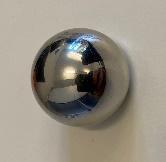 | N/A |
| Total hip (7+16+ceramic spacer) | 2734784 / 4263 /48 GG + hip | Zimmer biomet | Titanium alloy | no movement or deflectionexpected at 1.5 T, 3T, no information about heating | Femoral stem part, ceramic head and acetabular shell | 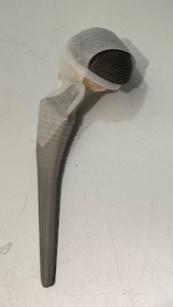 | 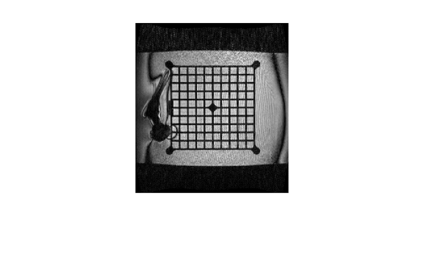 |
